# Serum levels of neurotensin, pannexin-1, and sestrin-2 and the correlations with sleep quality or/and cognitive function in the patients with chronic insomnia disorder

**DOI:** 10.3389/fpsyt.2024.1360305

**Published:** 2024-05-13

**Authors:** Ai-Xi Su, Zi-Jie Ma, Zong-Yin Li, Xue-Yan Li, Lan Xia, Yi-Jun Ge, Gui-Hai Chen

**Affiliations:** ^1^ Department of Neurology (Sleep Disorder), The Affiliated Chaohu Hospital of Anhui Medical University, Chaohu, China; ^2^ Department of General Medicine, the First Affiliated Hospital of Bengbu Medical University, Bengbu, China

**Keywords:** cognition, insomnia disorder, neurotensin, pannexin-1, sestrin-2

## Abstract

**Objectives:**

To examine serum concentrations of neurotensin, pannexin-1 and sestrin-2, and their correlations with subjective and objective sleep quality and cognitive function in the patients with chronic insomnia disorder (CID).

**Methods:**

Sixty-five CID patients were enrolled continuously and fifty-six good sleepers in the same period were served as healthy controls (HCs). Serum levels of neurotensin, pannexin-1 and sestrin-2 were measured by enzyme-linked immunosorbent assays. Sleep quality was assessed with the Pittsburgh Sleep Quality Index (PSQI) and polysomnography, and mood was evaluated by 17-item Hamilton Depression Rating Scale. General cognitive function was assessed with the Chinese-Beijing Version of Montreal Cognitive Assessment and spatial memory was evaluated by Blue Velvet Arena Test (BVAT).

**Results:**

Relative to the HCs, the CID sufferers had higher levels of neurotensin (*t*=5.210, *p*<0.001) and pannexin-1 (*Z*=−4.169, *p*<0.001), and lower level of sestrin-2 (*Z*=−2.438, *p*=0.015). In terms of objective sleep measures, pannexin-1 was positively associated with total sleep time (*r*=0.562, *p*=0.002) and sleep efficiency (*r*=0.588, *p*=0.001), and negatively with wake time after sleep onset (*r*=−0.590, *p*=0.001) and wake time (*r*=−0.590, *p*=0.001); sestrin-2 was positively associated with percentage of rapid eye movement sleep (*r*=0.442, *p*=0.016) and negatively with non-rapid eye movement sleep stage 2 in the percentage (*r*=−0.394, *p*=0.034). Adjusted for sex, age and HAMD, pannexin-1 was still associated with the above objective sleep measures, but sestrin-2 was only negatively with wake time (*r*=−0.446, *p*=0.022). However, these biomarkers showed no significant correlations with subjective sleep quality (PSQI score). Serum concentrations of neurotensin and pannexin-1 were positively associated with the mean erroneous distance in the BVAT. Adjusted for sex, age and depression, neurotensin was negatively associated with MoCA score (*r*=−0.257, *p*=0.044), pannexin-1 was positively associated with the mean erroneous distance in the BVAT (*r*=0.270, *p*=0.033).

**Conclusions:**

The CID patients had increased neurotensin and pannexin-1 and decreased sestrin-2 in the serum levels, indicating neuron dysfunction, which could be related to poor sleep quality and cognitive dysfunction measured objectively.

## 1 Introduction

Insomnia is the most common sleep problem in the general and clinical population. The patients who meet with the diagnostic criteria for insomnia of ICSD-3 (1) complain about significant daytime symptoms such as fatigue, distracting attention, deteriorating memory and mood disorders (2, 3). Among these symptoms, memory impairment is the most common complaint of daytime dysfunction which affects the individual’s social and/or professional ability and other social functions to a certain extent (4). Evidence-based medicine shows that the risk of memory impairment increases significantly with the increase in the severity of insomnia (5).

At present, the researches on the brain mechanism of memory impairment in chronic insomnia patients mainly focuses on the macro level. Macroneuroimaging findings in insomnia patients have shown that structural changes in brain regions associated with memory formation (6). For instance, compared to healthy people, patients with chronic insomnia disorder (CID) displayed smaller hippocampal volume (7) and larger gray matter volume in the left caudate nucleus (8) with the voxel-based morphometry. In addition, CID patients had decreased thalamic connectivity with the left amygdala, parahippocampal gyrus, putamen, pallidum and hippocampus across the whole sleep phase (9). These results suggested an impairment of brain structures and neuronal function, in which some were highly associated with spatial memory, exists in the patients with CID. Due to its importance in daytime function, spatial memory has been paid more and more attention by neuropsychological researchers. Sleep can promote the stabilization of initially labile spatial memory traces and integrate them into neocortical networks for long-term storage (10). Numerous animal (11) and human (12, 13) studies have shown that various forms of experimental sleep deprivation could induce significant deficits in spatial memory performance.

Although neuroimaging method can objectively show the brain macrostructure of the subjects, it’s costly and skeptical because of difficulty in comparing the results of repeated tests. Over the last decade, several key technological advanced, including calcium imaging (14), optogenetic (15), and chemogenetic manipulations (16), have made the relationship between brain microstructure and cognition in the field of sleep research become emerging. However, these technologies were only suitable to animal studies. The acquisition of microcosmic brain indicators (neurotransmitters, neuromodulators, peptides, neuromolecules, etc.) in human serum overcomes the defects. So more and more researchers arouse interest in microcosmic indicators.

Nerve cells link to each other through molecular channels and gap connections to form functional networks (17). When some microscopic damage (mitochondrial dynamic imbalance, free radical production, calcium accumulation, etc.) occurs to neurons, it will lead to a series of pathological changes (18). Therefore, the structural and functional integrity of neurons is an interesting and meaningful field to be explored. Nevertheless, the research in patients with CID is scarce.

As far as we know, only few groups have studied the changes of microscopic neuronal structural markers in patients with CID. The results showed that the serum levels of neurofilaments heavy chain and light chain [NfH and NfL, structural elements located in the axon endings of neurons (19)], and neuron-specific enolase [NSE, a neurotropic factor which localize mainly in the cytosol of neuronal cells (20)] increased and the changed levels of these proteins were correlated with insomnia severity and cognition impairment (21). Furthermore, after long-term effective therapy, their serum levels were significantly reduced and even equivalent to those of healthy sleepers. This indicated CID was related to neuronal damage, and which might be connected to abnormal cognitive function to some extent. Research in this area needs to be expanded and deepened in order to better understand the neuronal mechanism of CID.

Neurotensin, a group of neuropeptides containing 13 amino acids, is widely distributed in the brain and regulates various physiological activities including metabolism, thermoregulation, reward mechanism and regulation of sleep-wake cycles (22). One research has shown that selective activation of neurotensin neurons in lateral hypothalamus elicited rapid transitions from non-rapid eye movement (NREM) sleep to wakefulness and produced sustained arousal with higher locomotor activity (23). As a neuromodulator, neurotensin also can modulate cognitive processes (24). In physiological condition, neurotensin induces the glutamate transmission in brain cognitive areas, leading to increased synaptic plasticity, which facilitates learning and memory processes (25). To sum up, we consider that neurotensin may be involved in dysfunction associated with insomnia and abnormal cognition under condition of CID.

Pannexin-1, a gap junction protein, is widely expressed in central neurons and astrocytes, and its pathophysiological activity is highly related to neurons (26, 27). It was found that inhibition of pannexin-1 significantly alleviated neuronal apoptosis and degeneration in rats with intracerebral hemorrhage (28). In recent years, researchers have found that pannexin-1 was a potential new player in the regulation of cerebral homeostasis during sleep-wake cycle, via an indirect effect of released ATP on adenosine receptors and through interaction with other somnogens (29). It has been reported that pannexin-1 modulated the induction of excitatory synaptic plasticity under physiological contexts and contributes to neuronal death under inflammatory conditions (30). Moreover, it was found that an age-dependent increase in the pannexin-1 expression correlated with increased amyloid-β (Aβ) levels, which was thought to be the major cause of the cognitive deficits in Alzheimer’s disease (AD) (31). Therefore, we can assume that the overexpression of pannexin-1 may lead to synaptic dysfunction and thus affect cognitive dysfunction.

In addition to direct damage, when the protective factors of neurons *in vivo* are weakened, abnormal neuron function will also occur, leading to some physiological and pathological changes. Sestrin-2, a pressure sensor protein, was found to have a neuroprotective effect by polarizing microglia cells in the ischemic mouse brain from the pro-inflammatory M1 to the anti-inflammatory M2 phenotype (32). By augmenting sestrin-2 expression in disease models, the function of neurons was enhanced, allowing them to cope with oxidative stress and thus adapt to stressful conditions (33). A previous study has shown that patients with primary insomnia had a reduced ability to resist oxidative stress (34). Meanwhile, excessive oxidative stress may link to decreased neurite branching, learning and memory in a neurodevelopmental disorder (35). Sestrin-2 acts as an antioxidant, the changed concentration of it in patients with CID and its relationship with sleep quality and cognitive function are worth exploring.

The above three markers can be obtained from serum (22, 36), and it has not been studied whether serum levels of neurotensin, pannexin-1 and sestrin-2 are related to sleep quality and cognitive function in patients with CID. The aims of this study were to explore (1) whether the serum levels of neurotensin, pannexin-1 and sestrin-2 change in the patients with CID; (2) whether altered serum levels of these neuron-related markers correlated with poor sleep quality; and (3) the relationship between these serum biomarkers and cognitive function in the patients with CID.

## 2 Materials and methods

### 2.1 Subjects

A total of 121 people, consisting of 65 subjects with CID and 56 healthy controls (HCs), were included in the study. All enrolled subjects were recruited from the Clinic of Sleep Disorders in the Affiliated Chaohu Hospital of Anhui Medical University. They should meet the following criteria: (1) meeting the criteria in the third edition of the International Classification of Sleep Disorders (37); (2) age is 18~60 years old; (3) 6 years of education or above, with normal understanding of the items asked; (4) not experiencing any medical diseases (including immunologic, endocrine, cardiovascular, neurologic, liver, or kidney diseases), neurological diseases and psychiatric disorders; (5) not taking sedatives, antidepressants, antipsychotics, or any other drugs within 4 weeks; (6) non-pregnant or non-lactating females. HCs recruited from the physical examination centers at the same hospital were chosen as the control group using the following criteria: (1) no complaints or history of insomnia or mood disorders; (2) age is 18~60 years old; (3) 6 years of education or above, with normal understanding of the items asked; (4) not experiencing any medical diseases (including immunologic, endocrine, cardiovascular, neurologic, liver, or kidney diseases), neurological diseases and any psychiatric disorders; (5) total score < 7 on the Pittsburgh Sleep Quality Index (PSQI) (38); (6) total score < 7 on the 17-item Hamilton Depression Rating Scale (HAMD-17) (39); and (7) Montreal Cognitive Assessment, Chinese-Beijing Version (MoCA-C) score ≥ 26 (40).

The study was approved by Clinical Trial Ethics Committee of the Affiliated Chaohu Hospital of Anhui Medical University (No.: KYXM− 202108–005) and was conducted in accordance with the principles of the Declaration of Helsinki.

### 2.2 Baseline data collection

Information on demographic characteristics [i.e., age, sex, body mass index (BMI) and educational information], medical history, and family history were collected using a questionnaire developed by our group.

### 2.3 Assessment of mood states

The HAMD-17 was used to assess depression severity (39). The maximum score on the HAMD-17 is 52. A higher total score indicates greater severity of depression symptom. The HAMD-17 score cut-offs are as follows: ≤ 7, no depression; ≥ 8, mild depression; ≥ 18, moderate depression; and ≥ 24, severe depression (41).

### 2.4 Evaluations of subjective and objective sleep

The PSQI is designed to measure subjective quality of sleep over the past month (38). It measures seven components of sleep: latency, quality, duration, disturbances, efficiency, the use of sleep medications, and daytime dysfunction. A higher total score indicates worse sleep quality. In a study of Chinese community groups, it is found that PSQI score ≥ 7 could sensitively distinguish poor sleepers and healthy sleepers (42).

The polysomnography (PSG) is used to collect objective sleep data. The data is used to assess sleep continuity and structure. In our study, the former indicators include total sleep time (TST), numbers of arousals (NA), sleep efficiency (SE), wake time (W) and sleep onset latency (SOL); the latter include wake-time after sleep onset (WASO), rapid eye moment (REM) sleep latency (REM-SL), time (REM) and percentage (REM%) of REM sleep, time (N1) and percentage (N1%) of sleep stage 1, time (N2) and percentage (N2%) of sleep stage 2, and time (N3) and percentage (N3%) of slow wave sleep (SWS) in the non-REM sleep. Sleep parameters were staged according to the 2012 American Academy of Sleep Medicine Manual for the Scoring of Sleep and Associated Events recommendations (43). Due to the complexity of PSG operation, the compliance of some subjects was affected. Finally, 29 patients with CID and 25 healthy sleepers voluntarily completed the PSG detection.

### 2.5 Evaluations of cognitive function

#### 2.5.1 General cognition

MoCA-C was used to evaluate the general cognitive abilities of subjects. The maximum score for the MoCA-C is thirty, however, the score < 26 indicates the cognitive impairment in China (44).

#### 2.5.2 Spatial memory

**Figure 1:**
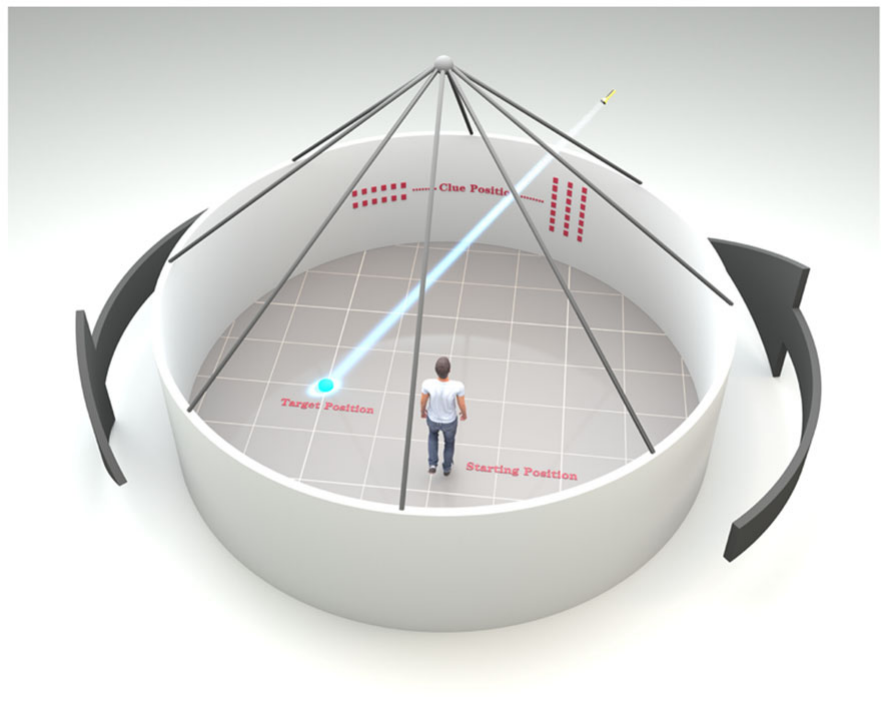
Schematic diagram of blue velvet arena test.

A modified version of the Blue Velvet Arena Test (BVAT) was used to evaluate spatial memory (45). BVAT is a human spatial navigation test that simulates the principles of the rodent Morris Water Maze experiment (the most widely used hippocampus-dependent spatial learning and memory task) (46). The modified version simplifies the procedure and allows for greater participation. In the middle of a moderate room with well light and obvious visual signs, we laid an equipment which consists of a 2.9-m diameter and 2.8-m height tent covered with blue velvet and eight curtains spaced 45 apart. The tents between the intervals were the same to prevent subjects from getting the influencing results according to the appearance cues. At a height of 1.5-m from the ground, two horizontal stripes and three vertical stripes were randomly set on the curtain as directional signs. A 12-cm diameter hidden laser lamp was located on top of the tent. When the subject entered the tent, its position was the starting position, the position irradiated by the laser light on the ground was the target position, and the horizontal and vertical stripes on the curtain were the clue positions. The operator helped subject remember the relative relationship between the starting position, the target position, and the clue positions. After the subject was told to step out of the tent with his back to the tent, the target laser lamp position was hidden and the tent was rotated randomly. After the rotation stopped, the subject was asked to accurately find the target position from the starting position according to the guidance of the clue position. After the subject confirmed the target position he had found, the operator placed the small flag on the target position. At this time, the target laser lamp position on the ground show again, and the subject was asked to readjust the relative relationship between the starting position, the clue positions and the target position, and then the next test was conducted for a total of 8 times. Finally, the errorous distance between the location of the small flag and the hidden real target position in each test was calculated, and the average value was taken for 8 times, namely, the average errorous distance. The greater the value, the greater the distance away from the correct position and the worse the navigation ability of the subject (**Figure 1**).

### 2.6 Serum collection

After the intervention and all other assessments have been completed, a 3-mL sample of venous blood was obtained from each subject the next morning between 7:30 a.m. and 8:00 a.m., with fasting and avoidance of strenuous activity and mental stimulation. The sample was centrifuged at 3000 rpm for 5 min and then stored with anticoagulant and frozen at −80°C. Serum concentrations of neurotensin, pannexin-1 and sestrin-2 were measured using enzyme-linked immunosorbent assay (Jianglai biotechnology, Shanghai, China).

### 2.7 Statistical analysis

SPSS 26.0 statistical software was used for statistical processing of the research data. Normally distributed data were expressed as mean ± standard deviation, and student’s *T*-test was used for comparison between groups. The non-parametric data were expressed as the 25th, 50th and 75th percentiles [*P*_50_ (*P*_25_, *P*_75_)], and comparison between groups was analyzed by Mann-Whitney *U* test. Spearman’s correlation analysis was used to analyze the correlation between serum markers and sleep quality and cognitive function, respectively. Partial correlation analysis was used to analyze correlations between serum biomarkers and sleep parameters and PSQI (adjusted for sex, age, and HAMD) and between serum biomarkers and general cognitive function and spatial memory (adjusted for sex, age, and HAMD). Receiver operating characteristic (ROC) curve was constructed and area under the receiver operating curve (AUC) was calculated to evaluate the diagnostic sensitivity and specificity of biomarkers. Two-sided *P* values ≤0.05 were considered statistically significant.

## 3 Results

### 3.1 General information

There were no significant differences in sex, age, BMI and education years (*P >*0.05, **Table 1**) between the CID and the HCs. However, the patients with CID had significantly higher HAMD-17 scores compared to the HCs (*P* < 0.001, **Table 1**). Most patients with CID have emotional problems. But for our study, CID group only had mild depression and could not be diagnosed as depressed according to DSM-5 (47).

**Table 1:** General demographic data and depression status of enrolled subjects.

Terms	CID	HCs	Statistics	*P*
Number of cases	65	56		
Disease Duration(months)	26.3	/		
Male/Female	19/46	22/34	*χ* ^2^ = 1.358	0.244
Age (years)	47.0 (41.5, 51.0)	41.0 (36.0, 49.8)	*Z*=−1.834	0.067
Education (years)	9.0 (6.0, 12.0)	9.0 (6.0, 12.0)	*Z*=−1.307	0.191
BMI (kg/m^2^)	22.7 (20.6, 24.1)	22.2 (20.0, 24.0)	*Z*=−0.993	0.321
HAMD-17 (score)	9.0 (6.0, 12.0)	0.5 (0.0, 1.8)	*Z*=−8.989	< 0.001

CID, chronic insomnia disorder; HCs, healthy controls; BMI, body mass index; HAMD-17, Hamilton Depression Rating Scale-17.

### 3.2 Sleep quality

The PSQI score in the CID group (*P*<0.001, **Table 2**) was significantly higher than that in the HCs. PSG results showed the patients with CID had significantly lower TST, N3, N3%, and SE, and significantly longer SOL, WASO, and W compared to the HCs, (*P*<0.05, **Table 2**). There were no significant differences in other sleep parameters between the two groups (*P*>0.05, **Table 2**). These results indicated that the patients who were recruited into the study exactly met our inclusion criteria.

**Table 2:** Comparison of sleep quality between the two groups.

Categories of tasks	Terms	CID	HCs	Statistics	*P*
Subjective sleep	PSQI (score)	14.0 (12.0, 16.0)	0.0 (0.0, 1.0)	*Z*=−9.562	<0.001
Objective Sleep	TST (min)	405.2 ± 61.0	441.0 ± 34.6	*t*=−2.701	<0.05
	NA (times)	19.0 (16.0, 24.5)	18.0 (12.5, 21.0)	*Z*=−1.295	0.195
	WASO (min)	69.5 (53.0, 97.8)	31.0 (16.5, 57.0)	*Z*=−4.104	<0.001
	SE (%)	79.3 ± 10.6	90.3 ± 4.5	*t*=−5.089	<0.001
	SOL (min)	15.0 (7.0, 26.3)	7.5 (4.5, 15.0)	*Z*=−2.204	<0.05
	REM-SL (min)	112.5 (62.0, 187.3)	82.0 (65.0, 121.0)	*Z*=−0.885	0.376
	W (min)	95.5 (64.5, 140.5)	44.5 (26.0, 68.5)	*Z*=−4.502	<0.001
	REM (min)	71.7 ± 32.7	81.9 ± 23.8	*t*=−1.326	0.191
	REM%	18.1 ± 7.4	18.5 ± 4.8	*t*=−0.231	0.818
	N1 (min)	55.4 ± 29.5	43.6 ± 19.3	*t*=1.707	0.094
	N1%	12.9 (8.7, 16.2)	10.0 (7.2, 13.9)	*Z*=−1.865	0.062
	N2 (min)	208.0 (174.3, 272.5)	235.0 (217.5, 250.8)	*Z*=−0.729	0.466
	N2%	57.6 ± 13.7	53.4 ± 6.5	*t*=1.478	0.147
	N3 (min)	40.5 (7.5, 64.5)	73.5 (54.8, 96.5)	*Z*=−3.818	<0.001
	N3%	10.3 (1.8, 14.9)	17.2 (12.9, 22.2)	*Z*=−3.428	<0.01

Normally distributed variables are shown as mean ± SD; non-normally distributed variables are shown as P50 (P25, P75).

CID, chronic insomnia disorder; HCs, healthy controls; PSQI, Pittsburgh Sleep Quality Index Scale; TST, total sleep time; NA, numbers of arousals; WASO, wake-time after sleep onset; SE, sleep efficiency; SOL, sleep onset latency; REM-SL, rapid eye moment sleep latency; W, wake time; REM, rapid eye movement sleep time; REM%, percentage of rapid eye movement sleep time; N1, sleep stage 1; N1%, percentage of the sleep stage 1; N2, sleep phase 2; N2%, percentage of the sleep stage 2; N3, sleep stage 3; N3%, percentage of the sleep stage 3.

### 3.3 Cognitive function and serum biomarkers in CID

**Figure 2:**
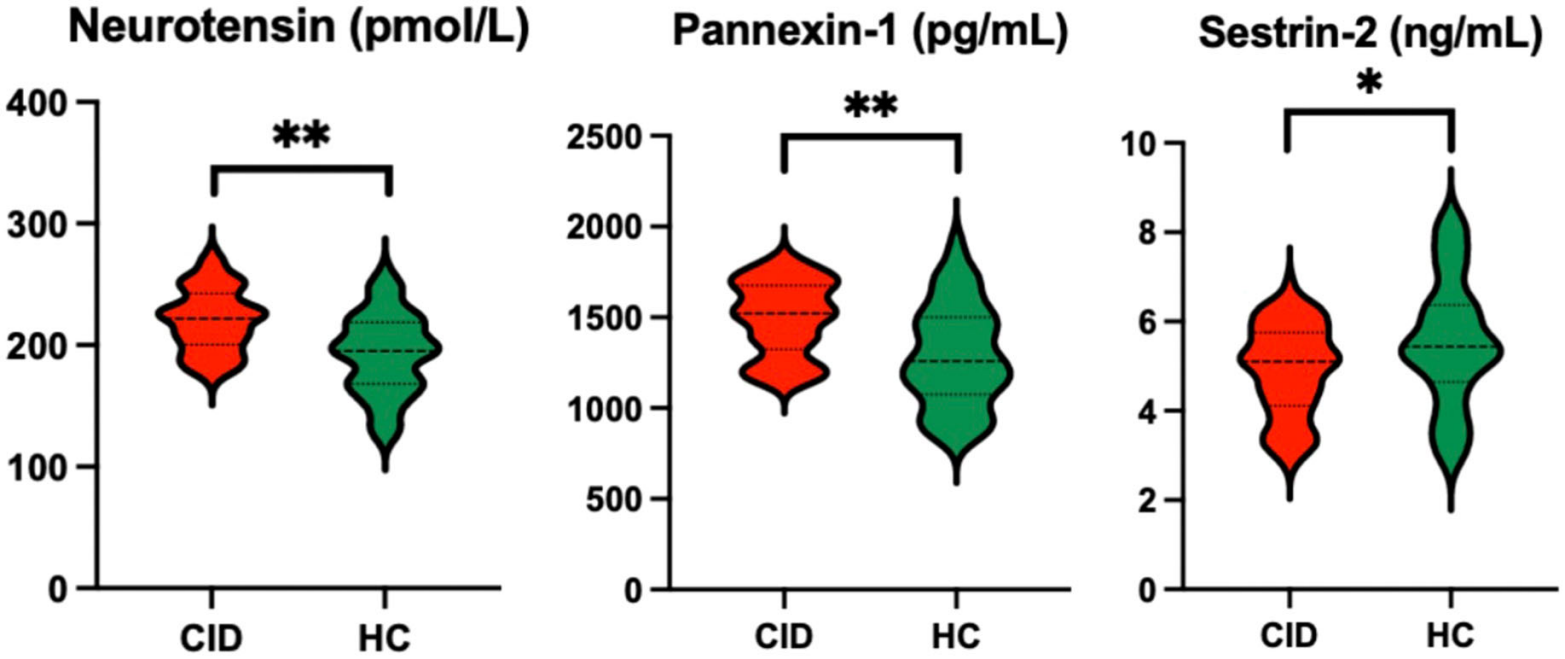
Comparison of serum biomarker levels between CID patients and HCs *P < 0.05 and **P < 0.01. CID, chronic insomnia disorder; HCs, healthy controls.

The patients with CID had significantly lower MoCA-C scores (*P* < 0.001) and longer average erroneous distance (*P* < 0.001) in BVAT than the HCs (**Table 3**). The CID patients had significantly higher serum levels of neurotensin, pannexin-1 (*P* < 0.001), and lower serum level of sestrin-2 (*P* < 0.05) compared to the HCs (**Table 3**, **Figure 2**).

**Table 3:** Comparison of cognitive function results and serum biomarkers concentration between the two groups.

	CID (*n*=65)	HCs (*n*=56)	Statistics	*P*
MoCA-C (scores)	24.0 (21.5, 27.0)	27.0 (26.0, 28.0)	*Z*=−4.380	<0.001
Average erroneous distance (cm)	22.8 (18.8, 26.8)	18.7 (16.6, 20.7)	*Z*=−4.424	<0.001
Neurotensin (pmol/L)	220.7 ± 25.9	192.7 ± 33.1	*t*=5.210	<0.001
Pannexin-1 (pg/mL)	1521.3 (1323.7, 1675.5)	1258.9 (1076.4, 1501.0)	*Z*=−4.169	<0.001
Sestrin-2 (ng/mL)	5.1 (4.1, 5.8)	5.4 (4.6, 6.4)	*Z*=−4.169	0.015

Normally distributed variables are shown as mean ± SD; non-normally distributed variables are shown as P50 (P25, P75).

CID, chronic insomnia disorder; HCs, healthy controls; MoCA-C, Chinese-Beijing Version of Montreal Cognitive Assessment.

### 3.4 Correlations between serum biomarkers, sleep parameters, and cognitive performance

Spearman correlations between serum biomarkers, sleep parameters and cognitive function are listed in **Table 4**. The serum concentration of pannexin-1 was positively correlated with TST (*r* = 0.562, *P* < 0.01) and SE (*r* = 0.588, *P* < 0.01), negatively correlated with WASO(*r* =−0.590, *P* < 0.01) and W (*r* =−0.582, *P* < 0.01). The serum concentration of sestrin-2 was positively correlated with R% (*r* = 0.442, *P* < 0.05) and negatively correlated with N2% (*r* =−0.394, *P* < 0.05). There were no significant correlations between serum neuron-related marker levels and PSQI scores (*P* > 0.05). Spearman correlation analysis showed that serum neurotensin and pannexin-1 levels were positively correlated with average erroneous distance in BVAT (*r* = 0.263, *P* < 0.05; *r* = 0.328, *P* < 0.01). There were no significant correlations between serum neuron-related marker levels and MoCA-C scores (*P* > 0.05).

**Table 4:** Correlation between serum marker levels with sleep quality and cognitive function in CID group.

Terms	Clinical parameters	Neurotensin	Pannexin-1	Sestrin-2
Subjective sleep quality (n=65)	PSQI (scores)	−0.001	−0.192	0.062
Objective sleep quality (n=29)	TST (min)	0.164	0.562^**^	0.018
	NA (times)	−0.118	−0.178	0.298
	WASO (min)	−0.212	−0.590^**^	−0.249
	SE (%)	0.285	0.588^**^	0.236
	SOL (min)	−0.123	−0.252	−0.319
	REMSL (min)	0.001	0.035	−0.182
	W (min)	−0.298	−0.582^**^	−0.268
	REM (min)	0.153	0.127	0.339
	REM%	0.031	−0.100	0.442^*^
	N1 (min)	−0.284	−0.093	0.277
	N1%	−0.293	−0.133	0.278
	N2 (min)	0.151	0.231	−0.216
	N2%	0.089	0.001	−0.394^*^
	N3 (min)	0.217	0.225	0.237
	N3%	0.268	0.141	0.246
Cognitive function (n=65)	MoCA-C (scores)	−0.071	−0.122	−0.135
	BVAT (cm)	0.263^*^	0.328^**^	−0.078

*P < 0.05 and **P < 0.01.

PSQI, Pittsburgh Sleep Quality Index Scale; TST, total sleep time; NA, numbers of arousals; WASO, wake-time after sleep onset; SE, sleep efficiency; SOL, sleep onset latency; REMSL, rapid eye moment sleep latency; W, wake time; REM, rapid eye movement sleep time; REM%, percentage of rapid eye movement sleep time; N1, sleep stage 1; N1%, percentage of the sleep stage 1; N2, sleep phase 2; N2%, percentage of the sleep stage 2; N3, sleep stage 3; N3%, percentage of the sleep stage 3; MoCA-C, Chinese-Beijing Version of Montreal Cognitive Assessment; BVAT, Blue Velvet Arena Test.

Partial correlations (adjusted for age, sex, and HAMD) between serum biomarkers, sleep parameters and cognitive performance are listed in **Table 5**. The serum concentration of pannexin-1 was positively correlated with TST (*r* = 0.496, *P* = 0.01) and SE (*r* = 0.501, *P* < 0.01), negatively correlated with WASO(*r* =−0.459, *P* < 0.05) and W (*r* =−0.427, *P* < 0.05). The serum concentration of sestrin-2 was negatively correlated with W (*r* =−0.446 *P* < 0.05). The serum concentration of neurotensin was negatively correlated with MoCA-C scores (*r* =−0.257, *P* < 0.05), and the serum concentration of pannexin-1 was positively correlated with average erroneous distance in BVAT (*r* = 0.270, *P* < 0.05).

**Table 5:** Partial correlation between serum marker levels and sleep quality and cognitive functionin in CID group (adjusted for sex, age and HAMD).

Terms	Clinical parameters	Neurotensin	Pannexin-1	Sestrin-2
Subjective sleep quality (n=65)	PSQI (scores)	0.053	−0.095	0.078
Objective sleep quality (n=29)	TST (min)	0.176	0.496**	0.090
	NA (times)	0.262	−0.040	−0.039
	WASO (min)	0.033	−0.459*	−0.374
	SE (%)	0.182	0.501**	0.385
	SOL (min)	−0.277	−0.302	−0.240
	REMSL (min)	0.059	0.104	−0.226
	W (min)	−0.176	−0.427*	−0.446*
	REM (min)	0.318	0.142	0.264
	REM%	0.209	−0.038	0.348
	N1 (min)	−0.120	−0.232	0.241
	N1%	−0.143	−0.345	0.187
	N2 (min)	0.097	0.249	−0.233
	N2%	0.011	0.009	−0.379
	N3 (min)	−0.009	0.255	0.234
	N3%	−0.021	0.183	0.265
Cognitive function (n=65)	MoCA-C (scores)	−0.257*	−0.245	−0.106
	BVAT (cm)	0.184	0.270*	−0.032

*P < 0.05 and **P < 0.01.

PSQI, Pittsburgh Sleep Quality Index Scale; TST, total sleep time; NA, numbers of arousals; WASO, wake-time after sleep onset; SE, sleep efficiency; SOL, sleep onset latency; REMSL, rapid eye moment sleep latency; W, wake time; REM, rapid eye movement sleep time; REM%, percentage of rapid eye movement sleep time; N1, sleep stage 1; N1%, percentage of the sleep stage 1; N2, sleep phase 2; N2%, percentage of the sleep stage 2; N3, sleep stage 3; N3%, percentage of the sleep stage 3; MoCA-C, Chinese-Beijing Version of Montreal Cognitive Assessment; BVAT, Blue Velvet Arena Test.

### 3.5 Correlation between illness’s duration and serum markers and spatial cognition

The correlations between illness’s duration and serum biomarkers and spatial cognitive function are listed in **Table 6**. Spearman analysis results showed that the duration was positively correlated with the serum concentration of pannexin-1 (*r* = 0.402, *P* < 0.01) and average erroneous distance in BVAT (*r* = 0.274, *P* < 0.05). Adjusted for age, sex, and HAMD, partial analysis results showed that the duration still was positively correlated with the serum concentration of pannexin-1 (*r* = 0.367, *P* < 0.05) and average erroneous distance in BVAT (*r* = 0.611, *P* < 0.001).

**Table 6:** Correlation analysis of disease duration (months) with serum markers and spatial cognitive function.

	Spearman analysis	Partial correlation analysis
Neurotensin(pmol/L)	0.074	0.017
Pannexin1(pg/mL)	0.402**	0.367*
Sestrin2(ng/mL)	0.116	0.077
BVAT(cm)	0.274*	0.611**

*P < 0.05 and **P < 0.01; BVAT, Blue Velvet Arena Test.

### 3.6 Diagnostic value of serum biomarkers

ROC curves of neurotensin, pannexin-1 and sestrin-2 were plotted to diagnose CID. The results showed that the AUC of neurotensin, pannexin-1 and sestrin-2 were 0.739, 0.720 and 0.371, respectively. Neurotensin and pannexin-1 can effectively distinguish CID patients from healthy sleepers with high sensitivity and specificity, and the cut-off values of neurotensin and pannexin-1 are 199.3 pmol/L and 1312.4 pg/mL, respectively (*P*<0.05, **Table 7**).

**Table 7:** Characteristics of potential serum biomarkers for CID in ROC analysis.

	AUC	Cut-off	Sensitivity	Specificity	95%CI
Neurotensin (pmol/L)	0.739	≥199.3	0.769	0.607	(0.651, 0.827)
Pannexin-1 (pg/mL)	0.720	≥1312.4	0.769	0.589	(0.628, 0.813)
Sestrin-2 (ng/mL)	0.371	≤3.2	0.969	0.054	(0.270, 0.472)

AUC, area under the receiver operating curve; CI, confidence interval.

## 4 Discussion

### 4.1 CID is associated with neuronal dysfunction

In this study, we found that serum levels of neurotensin, pannexin-1, and sestrin-2 were significantly different in patients with CID compared to the healthy controls. These results indicated a certain extent of neuronal dysfunction in patients with CID.

There were few studies on the structural markers of microscopic neurons in patients with CID until now. Our group previously found that the serum levels of neuron-related biomarkers (NfL, NfH and NSE) increase and significantly decrease after effective therapy. This result indicated that CID patients may have impairment of neurons structure. However, this viewpoint needs to be supported by further work.

Neurotensin is expressed abundantly throughout the brain. They can act through diverse intracellular signaling cascades to participate in neuronal activity by modulating activity of ion channels, ionotropic and metabotropic neurotransmitter receptors, and presynaptic release of neurotransmitters (48). In the patients of autism spectrum disorders (regarded as neurodevelopmental disorders), serum concentration of neurotensin is elevated and trigger mast cells to release inflammatory factors, which stimulate microglia proliferation and activation, leading to disruption of neuronal connectivity (49). We examined the levels of neurotensin in patients with CID and found increased serum levels compared to healthy controls. This is consistent with our expectation that patients with CID have neuronal dysfunction.

Pannexin-1 is found in neurons and various glial cells (astrocyte, microglia, oligodendrocytes), and the pathophysiological activity of pannexin-1 is highly related to neurons (50). Up-regulation of pannexin-1 expression has been demonstrated to exert an essential influence in multiple brain injury diseases such as epilepsy (51), sepsis-associated encephalopathy (52), and subarachnoid hemorrhage (53). Pannexin-1 channel inhibitor can improve neurological dysfunction and exert neuroprotective effects by means of reducing inflammasomes activation (54). In line with the results in human traumatic brain injury, serum pannexin-1 concentrations increases after intracerebral hemorrhage, hinting that serum pannexin-1 may be at least partially originated from central nervous system (55). In our study, the increase of serum pannexin-1 indicates that there exists neuronal damage in the patients with CID.

Neurons are particularly vulnerable to hypoxia due to their unique structure and function, such as the presence of unsaturated fatty acids, high metabolism, and dependence on oxygen (56). Sestrin-2 acts as an antioxidant which are expressed abundantly through the neurons of brain and can play a neuroprotective role in the absence of oxygen through various pathways (57, 58). In the hippocampal CA1 subfield of rats which are subjected to transient global ischemia-induced hippocampal neuronal injury, sestrin-2 expression is progressively increased (59). And the over-expression levels of sestrin-2 exhibit neuroprotection by shifting microglia polarization from the M1 to M2 phenotype in ischemic mouse brain. Interestingly, we found the serum sestrin-2 level was decreased in the patients with CID. This indicated that the above compensatory changes can no longer be produced in patients with CID. Moreover, patients with CID are likely to have brain damage represented by neuronal dysfunction, resulting in insomnia symptoms caused by reduced antioxidant stress ability.

### 4.2 Relationship between neuron-related markers and sleep quality

The role of neurotensin in sleep-wake cycle is being explored. A recent study has shown that locomotor activity decreased during both day and night, while sleep increased exclusively during the nighttime in neurotensin knockout zebrafish larvae, and this effect may be synergistic with melanin-concentrating hormone (MCH) neurons (60). MCH is a neuropeptide produced by neurons in the posterolateral region of the hypothalamus and is considered to be neuromodulating (61). These neurons diffuse throughout the central nervous system. Neurons identified as MCH do not fire during wake; they fire selectively during sleep, occasionally during slow wave sleep (SWS) and maximally during REM sleep (62). Microinjections of MCH into the ventrolateral preoptic area (VLPO, recognized as one of the key structures responsible for the generation of NREM sleep) significantly increased NREM sleep (47) and microinjection into the right locus coeruleus (LC, a noradrenergic nucleus that plays a key role in waking-related activities) facilitated REM sleep (63). These results indicated that MCH neurons inhibit the arousal system and/or activate the sleep system to promote sleep. Biochemical and structural studies have shown that neurotensin-receptor was expressed in MCH neurons and that neurotensin elimination increased MCH expression. These results suggested that neurotensin can promote arousal to a certain extent. Consistent with this, we found that serum neurotensin levels were higher in patients with CID than those in healthy individuals. Sleep loss in patients with CID may be caused by abnormal neurotensin expression due to damage to MCH neurons in the brain.

Pannexin-1 has also been found to be associated with arousal. A significant increase in wake percentage at the expense of a decrease in SWS was found in pannexin-1 knockout mice as compared to the control ones and this difference was especially pronounced during the 12-h dark period in the chamber. Previous studies showed that among various processes implicated in sleep homeostasis, adenosine—a critical component of the cell metabolic pathway—was a prominent physiological mediator of sleep homeostasis (64). Pannexin-1 is a release mechanism for mechanically stimulated adenosine release, pannexin channels can regulate rapid adenosine release and be targeted to differentially affect mechanically stimulated adenosine due to brain damage (65). Our previous studies have shown that the concentration of adenosine in CID patients was reduced and was associated with poor sleep quality (66). In the current study, objective sleep detection was performed on part of the population in our sample, we found that pannexin-1 was positively associated with TST, SE, and negatively associated with WASO and wake time. After controlling confounding factors such as sex, age and depression, there remained relevant. This seems to contradict the results of animal studies. We think this may be due to the fact that our sample is not large enough, or that the changes in serum pannexin-1 levels are not sufficient to have a significant arousal promoting effect on the central system.

Some diseases associated with sleep disorders were also found to be related to the occurrence of oxidative stress (67). Using genetic or pharmacological approaches to increase sleep in wild-type flies increases their resistance to oxidative stress, in the meantime, reducing oxidative stress in the neurons of wild-type flies by over-expression of antioxidant genes reduces the amount of sleep (68). This suggested that there was a bidirectional regulatory relationship between sleep and oxidative stress. Sestrin-2 is an antioxidant protein, and no studies have investigated the relationship between serum levels and sleep quality in patients with CID at present. We found that sestrin-2 was positively associated with the percentage of REM sleep and negatively associated with the percentage of N2 sleep. These results indicated that sestrin-2 was associated with objective sleep quality in patients with CID. However, after controlling confounding factors, we found that sestrin-2 was negatively associated with wake time. It means that the role of sestrin-2 in objective sleep is unclear, and a variety of confounding factors may involve in this process, which is still a worthy direction to explore.

Interestingly, there were no significant correlations between serum neuron-related marker levels (neurotensin, pannexin-1 and sestrin-2) and subjective sleep parameters (PSQI scores). This suggests that subjective and objective sleep appear to reflect two distinct neurophysiological processes.

### 4.3 Neuron-related markers were associated with cognitive impairment

Neurons play an important role in cognitive processes. Immunoinflammatory responses in neurons, altered synaptic plasticity between neurons, abnormal secretion of inter-synaptic neurotransmitters, changes in neurotrophic factors, and oxidative stress in neurons can all lead to abnormal physiological processes such as neuronal growth and differentiation, resulting in cognitive impairment (69, 70).

As a neuromodulator, neurotensin is widely present in the central nervous system and is involved in learning and reinforcement processes. Neurotensin has been proved to facilitate spatial and passive avoidance learning after microinject into the rat central nucleus of amygdala (regarded as an important role in regulating learning, memory and fear-related behaviors) (71).

Long-term enhancement (LTP) and long-term inhibition (LTD) in synaptic plasticity are generally considered to be the cellular biological basis of learning and memory (72). In hippocampal sections, disruption of pannexin-1 (through pannexin-1 gene knockout and pannexin-1 channel inhibition) enhanced neuronal excitability, promoted LTP induction, and hindered the induction of LTD. Furthermore, pannexin-1 knockout mice show impaired object recognition and spatial memory in the Morris water maze (73). It is suggested that pannexin-1 regulates the mechanism of hippocampal synaptic plasticity and plays a key role in learning and memory.

In recent years, the relationship between sestrin-2 and cognition has not been clearly verified. Studies have shown that the serum sestrin-2 protein over-expressed significantly in the AD group compared to mild cognitive impairment (MCI) and elderly control groups. A difference in serum sestrin-2 concentration between MCI and the control group is also evident (74). Aβ and Tau are two pathological proteins of AD. According to a recent study, the reduction in brain Aβ plaque may substantially slow cognitive and functional decline in patients with dementia or MCI due to AD (75). Interestingly, Aβ can induce sestrin-2 expression (57) and sestrin-2 is co-localized with phosphorylated-Tau immunoreactivity in a subset of neurofibrillary lesions (76). Based on these results, it is reasonable to suspect that cognitive impairment in AD patients may be caused by changes in sestrin-2 associated with two pathologic proteins (Aβ and Tau).

The results of the current study showed that serum neurotensin and pannexin-1 levels in the patients with CID were positively correlated with the spatial memory function assessed by BVAT. BVAT is a simulated navigation test of human real three-dimensional space, and it is a new spatial cognitive function detection method. The greater the average errorous distance of BVAT, the worse the spatial memory function of the subjects. When the distance was greater than 22.7 cm, the spatial memory function of the subjects can be considered as impaired (77). In addition, we found that after controlling confounding factors, the serum levels of neurotensin was negatively associated with MoCA-C scores and the serum levels of pannexin-1 was positively associated with average erroneous distance in BVAT. These results suggested that the changes in serum concentrations of neurotensin and pannexin-1 in patients with CID were associated with poor spatial memory function to some extent. What’s more, with the prolongation of insomnia duration, the level of pannexin1 in the serum of insomnia patients increased, and the spatial cognitive function impairment was more serious. However, this study did not reveal the relationship between sestrin-2 and cognition, which was consistent with our hypothesis. Sestrin-2 may not have an independent effect on cognitive function. It needs to interact with Aβ and/or Tau to cause cognitive impairment, which can only be achieved in AD patients. Abnormal serum sestrin-2 levels in patients with CID may be due to secondary changes resulting from neuronal damage.

### 4.4 Possible diagnostic and prognostic value of neuron-related markers

ROC curve analysis was conducted to assess the diagnostic value of these biomarkers for CID. The results showed that the AUC values of neurotensin and pannexin-1 were 0.739 and 0.720, respectively. The optimal cut-off values for serum neurotensin and pannexin-1 to identify patients with CID were 199.3 pmol/L and 1312.4 pg/mL, respectively. Therefore, we believed that neurotensin and pannexin-1 as objective indicators might be helpful for the diagnosis of CID. Combining these two indicators could provide a more sensitive way to distinguish CID from healthy sleepers.

However, the results of the study showed that the AUC value of sestrin-2 was only 0.371 and could not be used as an indicator for the diagnosis of CID. Therefore, we considered that the decreased ability to resist oxidative stress of brain and thus the weakened protective effect on neurons may need to cooperate with other neuronal injury mechanisms to jointly promote the occurrence and advancing of CID. People with poor resistance to oxidative stress are more likely to suffer from sleep disorders. In other words, the diagnostic value of sestrin-2 for CID could only be realized when oxidative stress reaches a certain severity/stage.

## 5 Conclusions

Neuronal damage may be a potential mechanism of insomnia. In addition, neurotensin and pannexin-1 are associated with spatial cognitive dysfunction in patients with CID. In the current study, the serological samples of neurotensin, pannexin-1, and sestrin-2 were obtained from the blood of subjects. At present, there is no clear research find that these three biomarkers can cross the blood-brain barrier (BBB), but according to the following three points, we can consider that the changed concentration in the serum samples we obtained from the peripheral blood can reflect the damage in the central nervous system. (1) One recent research has shown that sleep disruption can result in BBB dysfunction by generating new molecules such as free radicals and cellular metabolites and increasing vascular sheer stress, and alters cerebral perfusion (78). (2) In 2015, Jonathan Kipnis (79), and in 2020, Brant Weinstein (80), respectively, reported the presence of prox-1, vegfr-3 and lyve-1 positive lymphatic endothelial cells in mice and zebrafish dura, respectively, and these lymph vessels can effectively drain the brain spinal fluid and its substantial metabolic products to the deep cervical lymph nodes outside the brain, and eventually excrete into the blood system. (3) Although these three markers can come from other tissues, such as sestrin-2, can be found to be highly accumulated in muscle and liver, but in our study, we screened the subjects strictly, ruled out other diseases that might have impacts on the markers and conducted controlled health studies carefully.

## Data availability statement

The original contributions presented in the study are included in the article/Supplementary Material. Further inquiries can be directed to the corresponding authors.

## Ethics statement

The study was approved by Clinical Trial Ethics Committee of the Affiliated Chaohu Hospital of Anhui Medical University (No.: KYXM− 202108-005). The studies were conducted in accordance with the local legislation and institutional requirements. The participants provided their written informed consent to participate in this study.

## Author contributions

AS: Formal analysis, Investigation, Methodology, Visualization, Writing – original draft. ZM: Formal analysis, Investigation, Writing – original draft. ZL: Formal analysis, Investigation, Writing – original draft. XL: Project administration, Supervision, Writing – review & editing. LX: Project administration, Supervision, Writing – review & editing. YG: Methodology, Project administration, Supervision, Writing – review & editing. GC: Conceptualization, Funding acquisition, Methodology, Project administration, Resources, Writing – review & editing.
